# A Medico-Legal Case

**Published:** 1847-05

**Authors:** R. S. Strother, C. P. Mattingly

**Affiliations:** Bardstown, Kentucky; Bardstown, Kentucky


### ART. III.—*A Medico-legal Case.* Reported by R. S. STROTHER, M.D., and C. P. MATTINGLY, M.D., of Bardstown, Ky.

The following case was tried at the last term of the Nelson Circuit Court, and as the verdict of the jury turned entirely upon the medical testimony given it is deemed worthy of publication. In April, 1845, Mr. S., of this county, sold to Mr. M., of Jefferson, a negro man, warranting him to be sound; the man was then, and had been for six months, in jail. Three days after the sale he died. S. sued for his money; M. pleaded unsoundness in the negro and obtained a verdict in his favor. The following is a condensed statement of the facts in the case proved by a number of witnesses.

The man was from 40 to 45 years of age, of middle height, large head, short neck, broad chest, and fine muscular frame. With this conformation he was remarkably strong and active. Those who had known him for twenty years had never heard of his having lost a day from his work in consequence of sickness. In this high state of health he was incarcerated, and kept in confinement for six months. The jailor testified that, while in jail, he had complained of pain in his head and side, for which a physician had been consulted. The physician stated to the jury that he had twice visited the man in the six months, that he looked upon his indisposition each time as simply the result of costiveness, for which nothing was needed but gentle laxatives. At the expiration of the period he was sold as above stated, having a healthy appearance, considering himself well, and regarded as sound by all who saw him. He was taken from jail the day on which he was sold, and walked eleven miles without complaining of fatigue, keeping up with his master on horseback, and stopping to rest but once on the way. The day following he was put to labor at hard work, namely, ditching in a wet clayey soil, but was found incapable of executing a man's task. The next day he continued at the same work for several hours, but as he did not get on well his master, after threatening him for his indolence, put him to felling trees. While chopping later in the day he fell forward suddenly on his face, and, when the overseer who was close by went to him, was found insensible and speechless. His breathing was stertorous, and he frothed at the mouth. In this state he was carried to the house, and the family physician sent for. When Dr. L. arrived he pronounced the man in a dying condition; had his feet put into hot water; and attempted to draw blood, but none flowed. At 9 o'clock that evening he died, being about six hours from the time he fell down.

Thirteen hours after death Dr. L. made a partial examination of the body. He stated to the jury that he had examined the chest and abdomen, in which he found the following appearances: The heart was the smallest human heart he had ever seen, weighing, he thought, not more than four ounces, though he did not weigh it nor take it out of the body. One half was *tending to ossification*, and the other half was *mortified*, and so soft that he could have thrust his finger through it; he did not, however, cut into it, nor was he sure whether or not it contained any blood, but thinks it could not, as he did not *hear it rattle.* The percardium, according to the best estimate he could make, for he did not measure it, contained five gills of serum, and he informed the jury that any quantity over two drachms was evidence of disease. The smell of the cadaver was so offensive that all the persons in the house, "but himself, were made to *puke;* he was accustomed to such things."

The lungs he found in a still worse condition. In parts they were so mortified that they fell to pieces when not carefully handled. He did not take them out nor make an incision into them. The pleura of the lungs adhered firmly to the pleura of the ribs. He looked into the stomach, found it empty, of an unhealthy color, and "the folds gone." The alimentary canal gave strong evidence of disease, and the *mesenteric glands* were very much *enlarged*, being about the *size of summer grapes.* The liver was of a dark color, and had some blisters on it. He did not examine the brain, but thought he discovered cause enough for the death of the man in the thoracic and abdominal cavities. He took no notes of the examination, and was not assisted by any medical man; he has since become the family physician.

Dr. P., who until lately was Mr. M.'s physician, arrived shortly after the negro died, and, from the symptoms related to him, pronounced the case apoplexy. Dr. L. testified to the jury that he did not look upon apoplexy as a disease! The physicians of Bardstown and its vicinity concurred in the opinion of Dr. P. They could not believe that it was possible for a man laboring under extensive disease of the heart, lungs, and bowels, to have gone through the labor performed by this individual; and all the appearances revealed by the autopsy they regarded as *cadaveric* phenomena.

The jury, however, thought with the doctor who did not consider *apoplexy* a *disease*, and returned a verdict accordingly. The appearances presented by the dead body, as so vividly set forth by Dr. L. outweighed the testimony of all the other witnesses.

This case, it appears to us, is instructive in many ways. We submit it to the profession without the comments which naturally rise to the mind.

Bardstown, March 24th, 1847.

